# The efficacy and cost-effectiveness analysis of telerehabilitation for patients after arthroscopic ACL-reconstruction: a non-inferiority randomized controlled trial

**DOI:** 10.1186/s13018-025-06403-w

**Published:** 2025-12-24

**Authors:** Chenrui Yuan, Caiqi Xu, Lihua Huang, Yaohua He, Shengdi Lu

**Affiliations:** 1 Department of Sports Medicine, Shanghai Sixth People’s Hospital Affiliated to Shanghai Jiao Tong University School of Medicine, No 600 Yishan Road, Shanghai, China; 2 Department of Rehabilitation, Shanghai Sixth People’s Hospital Affiliated to Shanghai Jiao Tong University School of Medicine, Shanghai, China; 3 Department of Orthopedics, Shanghai Sixth People’s Hospital Affiliated to Shanghai Jiao Tong University School of Medicine, No 600 Yishan Road, Shanghai, China

**Keywords:** Anterior cruciate ligament reconstruction, Telerehabilitation, Cost-effectiveness analysis, Non-inferiority randomized controlled trial

## Abstract

**Purpose:**

This study aimed to assess the clinical and cost-effectiveness of telerehabilitation (TELE) compared to face-to-face rehabilitation (FTF) in patients following arthroscopic ACL reconstruction. Given the increasing demand for accessible rehabilitation methods, this trial investigates the feasibility of implementing TELE in a middle-income country like China.

**Methods:**

A prospective, non-inferiority randomized controlled trial (RCT) was conducted with 68 participants randomly assigned to either TELE or FTF rehabilitation. Participants were followed for 12 weeks, with assessments at baseline, 6 weeks, and 12 weeks after surgery. Primary outcomes included knee function (measured using the International Knee Documentation Committee Subjective Knee Form, IKDC). Secondary outcomes included pain, mobility, and functional status. Cost-effectiveness was analyzed using the incremental cost-effectiveness ratio (ICER).

**Results:**

Both groups showed similar improvements in clinical outcomes, with no significant differences in IKDC scores, pain levels, or range of motion at 12 weeks. However, TELE rehabilitation was significantly less expensive, with a total cost of 58,303.18 CNY compared to 82,358.90 CNY for FTF (*p* < 0.001). The ICER analysis demonstrated that TELE was a cost-effective alternative, with substantial cost savings per unit of effectiveness.

**Conclusion:**

Telerehabilitation may be a cost-effective and clinically comparable alternative to traditional face-to-face rehabilitation following ACL reconstruction. These findings may support the broader implementation of TELE in regions with limited access to in-person rehabilitation, especially in low- and middle-income countries like China. Further studies with longer follow-up periods are needed to confirm the long-term cost-effectiveness and health benefits of TELE.

**Trial registration:**

Chinese Clinical Trial Registry (https://www.chictr.org.cn/). Trial registration ChiCTR2100053313.

**Supplementary Information:**

The online version contains supplementary material available at 10.1186/s13018-025-06403-w.

## Background

The anterior cruciate ligament (ACL) is one of the key stabilizing structures in the knee joint and is also one of the most susceptible to injury. Studies show that ACL injuries are the most common findings in arthroscopic examinations following knee injuries [1–3]. Statistics from abroad indicate an incidence rate of about 60 cases per 10,000 people per year. The primary cause of ACL injuries is sports-related, accounting for over 70%, with the highest incidence in basketball and football players [1–3]. Additionally, ACL ruptures are more common among professional athletes in judo, wrestling, and track and field, as well as among amateurs in skiing, badminton, and volleyball [2]. Currently, most treatments involve arthroscopic ACL reconstruction using autografts or allografts [3, 4]. Follow-up study at 2, 3, and 5 years post-operation show that both grafts achieve satisfactory clinical outcomes in terms of knee stability, muscle strength, and postoperative activity levels, with no significant difference in clinical effectiveness between them [5]. Emerging regenerative approaches such as RegentK therapy have also been explored for ACL injuries, a recent scoping review identified multiple studies focusing exclusively on the conservative/regenerative RegentK treatment for ACL ruptures [6].

Postoperative rehabilitation training also determines the functional recovery after arthroscopic ACL surgery. Rehabilitation for the surgery can begin with education and guidance before the operation and should be implemented immediately after the surgery. The rehabilitation and training program of arthroscopic ACL reconstruction is a mature and standardized rehabilitation process.

Telerehabilitation refers to the delivery of rehabilitation medical services across regions using a combination of computer and communication technologies, remote sensing, remote control, and information processing technologies [7]. In telerehabilitation, therapists cannot physically touch patients, which limits techniques such as palpation, movement examination, guidance in movement training, and comprehensive observation [7, 8]. However, this can be mitigated with techniques used in telerehabilitation, such as wearable smart devices, assisting patients to adjust the camera for better observation, and using the patient’s own hands for palpation [7, 8].

Both telerehabilitation and traditional face-to-face rehabilitation can complete assessments and treatments, and are effective in health education and movement guidance. Telerehabilitation has certain advantages over face-to-face rehabilitation: (1) It breaks geographical barriers, providing convenience for patients/clients far from rehabilitation centers or with difficult commutes. (2) It offers convenience to patients/clients who find it extremely difficult to move or leave their homes due to injury or illness, such as those unable to use stairs post-surgery and without elevator access. (3) It benefits long-term home-based rehabilitation patients, reducing time and travel expenses. (4) It facilitates expert consultations (via remote video conferences). Studies from abroad indicate that telerehabilitation shows no significant difference from traditional offline rehabilitation in terms of treatment and postoperative recovery, and it can significantly save patients’ time while achieving the same rehabilitation and postoperative recovery results [7–9]. And Tedeschi et al. found telerehabilitation to be as effective as in-person physiotherapy in reducing pain and improving quality of life for patients with knee osteoarthritis [10].

Despite the methodological differences in studies and the health care system of various countries, understanding the clinical outcomes and the economic costs of telerehabilitation interventions may improve their efficiency. For example, aging populations and rising chronic disease burdens are driving up demand for rehabilitation services, while many health systems already face physiotherapist shortages; in this context, lower-cost telerehabilitation programs may therefore be needed to expand access to care and reduce financial strain on healthcare resources. The use of telerehabilitation in low and middle-income countries such as China is just emerging. As a result, data on the clinical and cost-effectiveness of telerehabilitation are scarce [11, 12]. To date, we are not aware of any study that has investigated the clinical and cost-effectiveness of physiotherapy using telerehabilitation in these countries. To study the clinical and cost-effectiveness of telerehabilitation, we developed a telerehabilitation-based intervention for patients after arthroscopic ACL reconstruction. This study, therefore, assessed the clinical and cost-effectiveness of telerehabilitation-based intervention (TELE) compared with in-clinic face-to-face rehabilitation (FTF) for patients after arthroscopic ACL reconstruction in China.

## Material and methods

### Study design

This was a prospective, two-armed, open-labelled, randomised, controlled, single-centre clinical trial. The assessors were blinded. This RCT adhered to the Consolidated Standards of Reporting Trials (CONSORT). It was conducted between November 2, 2022, and July 15, 2023 at the largest trauma center in Shanghai (Shanghai Jiao Tong University affiliated Sixth People’s Hospital). Just before hospital discharge, participants who underwent arthroscopic ACL reconstruction were randomly assigned to two groups: the in-home telerehabilitation group (TELE group) and the in-clinic face-to-face rehabilitation (FTF group). Participants received a 12-week intervention were followed up for 12 weeks. Participants were evaluated at baseline (before surgery), during the intervention (6 weeks after discharge), and at the end of intervention (12 weeks after discharge).

### Participants

The inclusion criteria were as follows: (1) patients aged 18 to 60; (2) diagnosed as unilateral primary ACL injury (may be combined with meniscus injury); (3) underwent unilateral arthroscopic ACLR surgery using autologous tendon; (4) participated in sports regularly; (5) normal access to smart devices.

The exclusion criteria were as follows: (1) patients with history of knee surgery on the affected side; (2) combined injury to other ligaments of the ipsilateral knee or (3) combined injury of contralateral knee (4) suffered from severe musculoskeletal disorders; (5) with existence of lower limb malformations.

All the participants signed an informed consent statement. This study was registered with the Chinese Clinical Trial Registry (No. ChiCTR2100053313) and was approved by the Ethics Review Committee of Shanghai Sixth People’s Hospital [No. 2022–045-(1)].

### Intervention

All participants received standardised rehabilitation training instructions, overseen by two trained physical therapists (see Supplementary Materials). Each therapist exclusively supervised one group to ensure consistency. The intervention’s intensity and duration adhered to standardized guidelines provided by a panel of experts. The intervention encompassed several components, beginning with an assessment both before and after the exercise regimen. This assessment involved a structured interview and observation to evaluate the patient’s progress. During each session, patients engaged in supervised exercises for approximately thirty minutes, focusing on mobility, strengthening, function, and balance.

The exercises’ intensity and difficulty level were individually tailored to each patient, taking into account their tolerance and specific needs. This approach ensured that each participant received tailored rehabilitation support.

#### Telerehabilitation group (TELE group)

The rehabilitation program was delivered through a smartphone APP (device: Joymotion app (Shanghai Medmotion Medical Management Co., Ltd., China)) which provided participants exercise instructions, feedback on their training performance, and the real-time two-way video and audio interaction with the PT. The APP was installed by a technician on the same day of the patient’s discharge. Internet connection was provided by the patients’ own home Wi-Fi. The physiotherapist at the rehabilitation center initiated weekly video sessions at scheduled times. The APP provides daily rehabilitation exercises with detailed instructions and records the exercise completion rates. The rehabilitation program was prescribed by the supervising PT and was assigned to the patient as “daily tasks”.

#### Face-to-face rehabilitation group (FTF group)

In the FTF group, after the participants were discharged, they were assigned to a rehabilitation clinic 3–4 times per week for 12 weeks after discharge to receive exercise guidance from physiotherapists face to face. The components of the intervention and following home exercises were prescribed according to PT’s assessment before and after exercise.

Importantly, aside from the delivery method, both groups followed the same standardized exercise protocol, the assigned PT in TELE group can only regulate frequency and times for each exercise, thereby minimizing bias and allowing a clear comparison of clinical outcomes and cost-effectiveness.

### Outcome measures

At baseline, the patients’ demographic and clinical characteristics, including comorbidities, were recorded. At each follow-up visit, cointerventions, health complications, adverse events, physical activity levels and patient-reported outcome measures (PROMs) were documented.

#### Primary outcome

The primary outcome was the IKDC (International Knee Documentation Committee) Subjective Knee Form score at 12 weeks. The IKDC is a validated and self-administered questionnaire designed for patients with a variety of knee disorders that assesses knee function, symptoms and ability to engage in sports activities, with a range from 0 to 87, in which 87 indicated no limitations in daily or sporting activities [13–15].

#### Secondary outcomes

Secondary outcome measures included the Lower Extremity Functional Scale (LEFS), 12-item Short Form Survey (SF-12), Numeric Pain Rating Scale (NPRS) during walking and knee range of motion (ROM) at 6 weeks and 12 weeks follow-up. The IKDC, LEFS, and knee ROM were used to evaluate knee function. The SF-12 is a general health questionnaire that includes 12 questions from each of the eight dimensions of the SF-36 [16]. It is designed to perform similarly to the SF-36 but requires less time for patients to complete [17]. The SF-12 consists of two subscales: the Mental Component Score (MCS-12) and the Physical Component Score (PCS-12). The scores are reported as Z-scores (differences compared to the population average, measured in standard deviations) and range from to 0 to 100 with an average of 50 points and standard deviation of 10 points [18, 19]. An MCS-12 score of 42 or lower may indicate clinical depression, while a PCS-12 score of 50 or lower is the recommended cut-off for the physical condition [20].

#### Cost measures

Costs included intervention and other healthcare costs, paid help at home, informal care, work absenteeism and presenteeism and unpaid productivity costs.

For estimating intervention costs, we collected data from Hospital information system (HIS) of Shanghai Jiao Tong University affiliated sixth people’s hospital and online payment system of Shanghai Medmotion Medical Management Company.

Other healthcare costs included costs related to the use of primary healthcare (eg, general practitioner), secondary healthcare (eg, hospital visits other than the initial hospital) and prescribed and over-the-counter medication. All these costs were collected from HIS.

Paid home care costs were assessed by asking participants to report the number of hours they received paid home care, which were valued by direct inquiry to patient at 6-week and 12-week follow-up. We also collected informal care costs from patients when they visited outpatient at follow-up timepoint. Informal care costs were collected through the total number of hours patients were received help from family, friends and other volunteers, and costs were calculated by multiply total hours and average hourly-income of Shanghai.

For estimating absenteeism and presenteeism costs, we used the Productivity Cost Questionnaire [21]. We valued the patients’ number of sickness absence days in accordance with the Friction Cost Approach (FCA; friction period = 12 weeks) using gender-specific price weights [19]. For presenteeism costs, we asked participants to report the total number of days that they went to work while experiencing health complaints and to report their performance level on these days on a scale ranging from 0 (not able to do anything) to 10 (able to do everything). Subsequently, we calculated the total number of presenteeism days using the following formula: Presenteeism days = ((10 − performance level)/10) * number of days with health complaints. Presenteeism days were valued using gender-specific price weights [22]. For estimating unpaid productivity costs, we asked participants to report the total number of hours they were unable to perform unpaid tasks (eg, chores, volunteer work and educational activities), which were valued using an average hourly-income of Shanghai [22].

#### Adherence and acceptability

Adherence was measured using several indicators: the TELE group participants’ reported completion of sessions, and the attendance rate of FTF group participants at sessions [23]. Additionally, both groups rated their agreement with statements concerning adherence and acceptability on a scale from 0 (‘strongly disagree’) to 10 (‘strongly agree’). Participants also provided qualitative assessments of their perceptions of the exercise protocol’s outcomes [23].

### Sample size, randomization and blinding

IKDC at 12 weeks after surgery was regarded as the primary outcome measure. The standard deviation of the pre-treatment and post-treatment IKDC scores was 11, the non-inferiority margin was set at 8.8 points (reflecting the IKDC’s minimal detectable change), the test level was α = 0.05, and the power was 1–β = 90% [14, 15]. Based on these assumptions, the required sample size was 54. Therefore, to account for up to a 20% withdrawal rate, a total of 64 patients were recruited.

Patients referred to Shanghai Jiao Tong University affiliated Sixth People’s Hospital suspected for ACL tear were informed about the study by the orthopaedic surgeon. At the second outpatient visit, after written informed consent, we randomized eligible patients to either telerehabilitation or in-hospital face-to-face rehabilitation using a central computer-generated randomization scheme in a 1:1 ratio with random blocks (maximum block size of six). Participants, physicians and physical therapists were not blinded.

### Statistical analysis

Patient data were coded and securely stored using the hospital’s electronic data capture system, hosted on local servers. The primary analysis focused on the per protocol population and the adverse events intention-to-treat (ITT) population. Sensitivity analyses were conducted using ITT population with multiple imputation. Baseline data were reported as mean ± SD with 95% confidence intervals (CIs) unless otherwise specified. Intergroup differences in baseline characteristics were evaluated with independent sample t-tests for continuous variables and Fisher’s exact test for categorical data. All data were analyzed using SPSS Statistics version 24.0 (IBM Corp, Chicago, IL) and R version 4.3.2 (R Foundation for Statistical Computing, Vienna, Austria).

Following treatment, continuous outcomes, including questionnaire scores and functional test results, were analyzed as changes from baseline. These outcomes were compared between intervention groups at each time point using a linear mixed model for repeated measures (nlme, version 3.1–163, in R), with changes from baseline as the primary metric. The model accounted for the interaction between time and intervention, adjusted for age and sex as fixed effects, and included participants as random effects. Differences between the least squares means of the groups were estimated at each time point (emmeans, version 1.9.0, in R). The gain in the TELE group was evaluated as noninferior only if intergroup mean difference and its one-sided 95% confidence interval (CI) were less than 8.8 IKDC points. On the basis of the methodology of a noninferiority randomized trial, we tested the null hypothesis (H0) of a group difference against the alternative that the two treatments are equivalent (H1) according to our noninferiority margin of 8.8 IKDC points. Results for each time point were reported as mean value with standard deviation (SD), and differences between groups were expressed as Coefficient with two-sided 95% confidence intervals (CIs).

The incremental cost-effectiveness ratio (ICER) was used to assess the cost-effectiveness of TELE group compared with FTF group. The ICER is the differential costs and outcomes between the TELE group and the FTF group. The numerator in the cost-effectiveness ratio is the monetary cost of the TELE intervention minus the monetary cost of PT. The annual costs of the projects were calculated by converting the 12-week costs, the period used for implementation. The denominator is the IKDC gained by TELE minus the IKDC gained by FTF at 12 weeks. Bootstrapping was used for a pair-wise comparison of the mean costs and effects between the TELE and FTF groups. CIs for the mean differences in effects were obtained by bootstrapping (1000 replications). The bootstrapped cost and effect pairs were also graphically represented on a cost-effectiveness plane [22].

## Results

### Participants

**Fig. 1:**
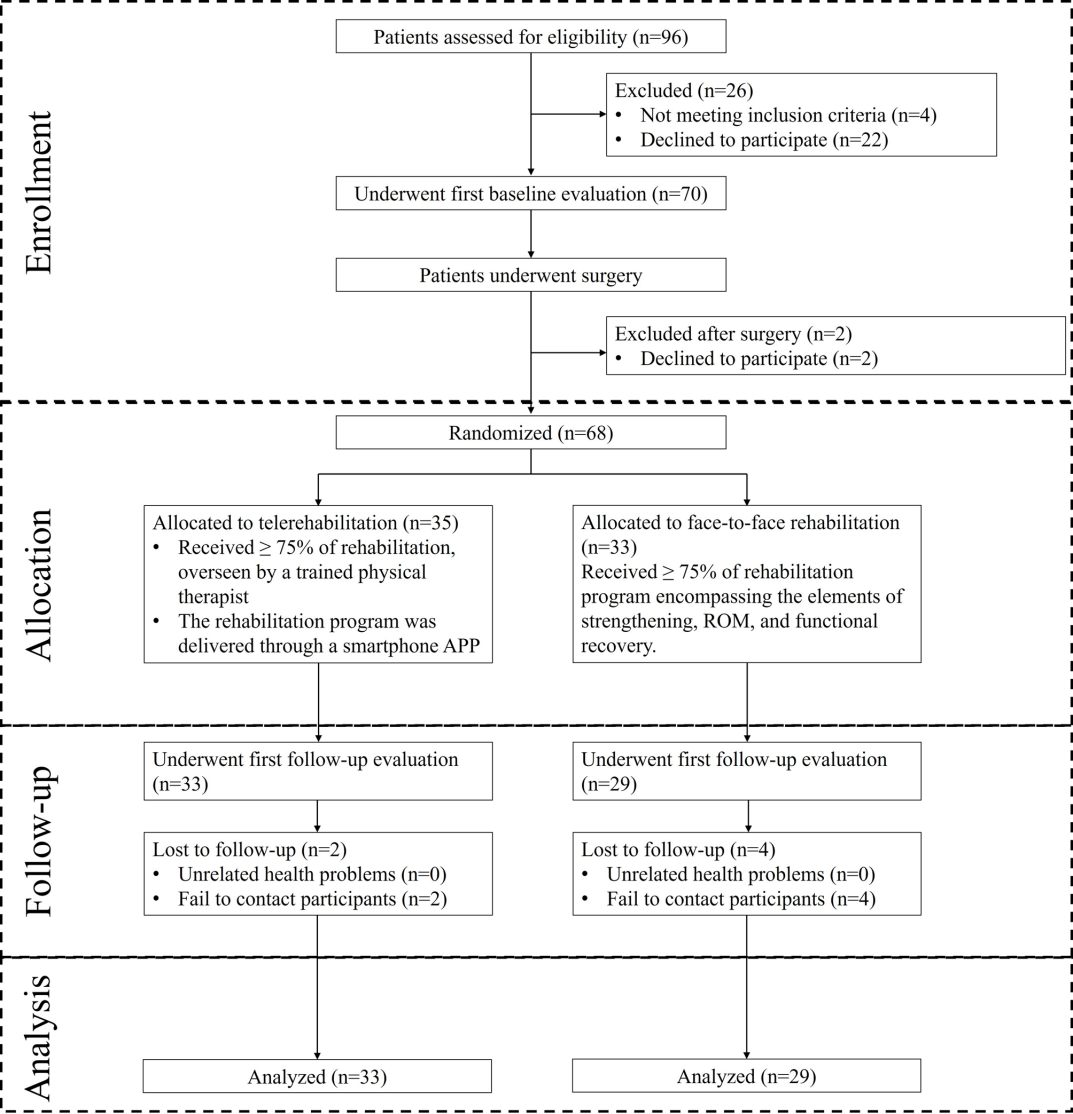
The flowchart of the study, from enrolment to analysis

We evaluated 96 patients with arthroscopic ACL reconstruction and randomized 68 into TELE (n = 35) and FTF (n = 33) rehabilitation groups. Participant demographics and baseline characteristics were similar between groups (see Table 1). Baseline outcome measures including IKDC scores, LEFS, SF-12 (PCS and MCS), NPRS, and measures of knee flexion, extension, and range of motion showed no significant differences between groups (Table 1). Figure 1 shows the flow diagram of the study.

**Table 1:** Baseline characteristics of the telerehabalitation group and face-to-face rehabilitation group

Sample characteristic	TELE group (N = 35)	FTF group (N = 33)	*P* value
Age (year)	29.14 (9.39)	31.09 (8.75)	0.380
Male patients (no. [%])	26 (74.29)	21 (63.64)	0.342
BMI (kg/m^2^)	25.46 (4.03)	25.01 (3.38)	0.627
Occupation (no.[%])			0.084
Manual worker	22 (66.67)	13 (44.83)	
Non-manual worker	11 (33.33)	16 (55.17)	
Education (no.[%])			0.345
Lower than high school	22 (62.86)	17 (51.52)	
High school and above	13 (37.14)	16 (48.48)	
Insurance type (no.[%])			0.970
Government	27 (81.82)	24 (82.76)	
Commercial	2 (6.06)	2 (6.90)	
Out-of-pocket	4 (12.12)	3 (10.34)	
Lachman test, positive (no.[%])	32 (96.97)	27 (93.10)	0.479
Previous menisus treatment (no.[%])	21 (63.64)	19 (65.52)	0.877
IKDC	50.71 (11.50)	54.09 (11.33)	0.227
IKDC (MAX adjusted to 100)	58.29 (13.22)	62.17 (13.02)	0.227
LEFS	56.49 (10.09)	57.64 (13.15)	0.686
SF-12 PCS	37.51 (6.04)	36.67 (7.46)	0.609
SF-12 MCS	48.88 (9.96)	52.48 (9.34)	0.129
NPRS	1.91 (1.40)	1.91 (1.59)	0.989
Active knee flexion (°)	123.57 (11.60)	124.79 (12.01)	0.672
Active knee extension (°)	1.21 (3.31)	0.86 (2.70)	0.653
Active ROM (°)	122.73 (12.69)	124.59 (13.74)	0.582
Passive knee flexion (°)	135.67 (12.13)	136.66 (12.78)	0.756
Passive knee extension (°)	− 2.12 (3.96)	− 2.59 (4.35)	0.661
Passive ROM (°)	137.79 (13.21)	139.24 (14.95)	0.686

TELE, telerehabalitation; FTF, face-to-face rehabilitation; BMI, body mass index; IKDC, International knee documentation Committee subjective knee form; LEFS, Lower extremity functional scale; SF-12, 12-item short form survey; PCS, physical component score; MCS, mental component score; NPRS, numeric pain rating scale; ROM, range of motion

### Primary and secondary outcomes

At the 12-week follow-up, the between-group difference in IKDC change was near zero: 0.15 (95% CI –5.81 to 6.11) (per-protocol n = 62, Table 3). The changes in outcomes for the TELE and FTF groups at 6 and 12 weeks after surgery are compared. At 6 weeks post-surgery, the IKDC score decreased by 5.39 points in the TELE group and by 9.24 points in the FTF group, with a *p*-value of 0.256, indicating no significant difference. By 12 weeks, both groups showed similar improvement, with an increase of around 8 points, and the *p*-value remained non-significant at 0.963. The LEFS scores showed minor decreases at 6 weeks, with no significant differences, and by 12 weeks, both groups improved (TELE by 9.15 points, FTF by 10.86 points), though this difference was not statistically significant (*p* = 0.560) (Tables 2, 3). Since the 95% CI for the IKDC difference falls within the predefined margin (± 8.8), TELE was statistically non-inferior to FTF.

**Table 2:** Changes in outcomes for the TELE and FTF groups at weeks 6 and 12 after surgery, per protocol analysis

Outcome	6 weeks post surgery			12 weeks post surgery		
	TELE group (N = 33)	FTF group (N = 29)	*P* value	TELE group (N = 33)	FTF group (N = 29)	*P* value
IKDC	− 5.39 (13.00)	− 9.24 (13.38)	0.256	8.15 (13.14)	8.00 (12.59)	0.963
IKDC (MAX adjusted to 100)	− 6.20 (14.94)	− 10.62 (15.37)	0.256	9.37 (15.10)	9.20 (14.47)	0.963
LEFS	− 4.12 (10.80)	− 3.31 (13.20)	0.791	9.15 (10.76)	10.86 (12.21)	0.560
SF-12 PCS	− 1.71 (7.96)	0.88 (7.26)	0.188	3.28 (8.89)	5.67 (8.80)	0.291
SF-12 MCS	0.19 (9.46)	− 1.86 (14.44)	0.506	3.31 (9.66)	3.37 (12.75)	0.983
NPRS	− 0.27 (1.82)	− 0.34 (1.84)	0.878	− 1.00 (1.50)	− 1.07 (1.46)	0.856
Active knee flexion (°)	− 11.45 (15.92)	− 16.14 (17.70)	0.277	2.58 (12.00)	− 0.28 (13.01)	0.373
Active knee extension (°)	2.73 (5.88)	3.97 (5.57)	0.400	− 0.15 (3.18)	− 0.17 (3.89)	0.982
Active ROM (°)	− 14.18 (19.24)	− 20.10 (21.02)	0.251	2.73 (12.63)	− 0.10 (14.78)	0.419
Passive knee flexion (°)	− 13.85 (17.57)	− 17.86 (17.70)	0.375	2.00 (13.76)	− 0.79 (13.20)	0.420
Passive knee extension (°)	1.97 (5.44)	2.93 (5.59)	0.496	0.15 (4.59)	0.34 (5.50)	0.881
Passive ROM (°)	− 15.82 (19.28)	− 20.79 (21.01)	0.335	1.85 (15.31)	− 1.14 (16.60)	0.464

TELE, telerehabalitation; FTF, face-to-face rehabilitation; IKDC, International knee documentation Committee subjective knee form; LEFS, Lower Extremity Functional Scale; SF-12, 12-item short form survey; PCS, physical component score; MCS, mental component score; NPRS, numeric pain rating scale; ROM, range of motion

**Table 3:** Effectiveness estimates from linear mixed effects models, per protocol analysis

Outcome	6 weeks post surgery			12 weeks post surgery		
	Coefficient	95% CI	*P* value	Coefficient	95% CI	*P* value
IKDC	3.847	(− 2.11, 9.81)	0.206	0.152	(− 5.81, 6.11)	0.960
IKDC (MAX adjusted to 100)	4.422	(− 2.43, 11.27)	0.206	0.174	(− 6.67, 7.02)	0.960
LEFS	− 0.811	(− 6.09, 4.47)	0.763	− 1.711	(− 6.99, 3.57)	0.525
SF-12 PCS	− 2.591	(− 6.43, 1.25)	0.186	− 2.398	(− 6.24, 1.44)	0.221
SF-12 MCS	2.050	(− 3.49, 7.59)	0.468	− 0.061	(− 5.60, 5.47)	0.983
NPRS	0.072	(− 0.72, 0.87)	0.859	0.069	(− 0.73, 0.87)	0.865
Active knee flexion (°)	4.683	(− 2.07, 11.44)	0.174	2.852	(− 3.91, 9.61)	0.408
Active knee extension (°)	− 1.238	(− 3.61, 1.14)	0.307	0.021	(− 2.35, 2.40)	0.986
Active ROM (°)	5.922	(− 2.03, 13.87)	0.144	2.831	(− 5.12, 10.78)	0.485
Passive knee flexion (°)	4.014	(− 3.14, 11.16)	0.271	2.793	(− 4.36, 9.94)	0.444
Passive knee extension (°)	− 0.961	(− 3.40, 1.48)	0.440	− 0.193	(− 2.64, 2.25)	0.877
Passive ROM (°)	4.975	(− 3.28, 13.23)	0.237	2.986	(− 5.27, 11.24)	0.478

IKDC, International knee documentation Committee subjective knee form; LEFS, lower extremity functional scale; SF-12, 12-item short form survey; PCS, physical component score; MCS, mental component score; NPRS, numeric pain rating scale; ROM, range of motion

In terms of SF-12 scores, the PCS slightly decreased for the TELE group at 6 weeks but increased by 12 weeks, although the difference compared to the FTF group was not statistically significant (*p* = 0.291 at 12 weeks). The MCS remained stable, with no significant difference between groups at either time point. Pain, as measured by the Numeric Pain Rating Scale (NPRS), decreased similarly in both groups at 6 and 12 weeks, with *p*-values of 0.878 and 0.856, respectively, indicating no significant difference in pain reduction. Measures of knee flexion, extension, and range of motion (both active and passive) also showed no significant differences between the groups at either 6- or 12-week post-surgery (Tables 2, 3).

Similarly, the LEFS and SF-12 scores showed no statistically significant differences between groups, with *p*-values ranging from 0.144 to 0.983 for various outcomes. Knee flexion and extension measures also did not show significant differences at either time point, as indicated by the non-significant *p*-values across the range of motion (ROM) measures. Overall, the models suggest no significant differences in effectiveness between the TELE and FTF groups for any of the assessed outcomes at either 6- or 12-week post-surgery (Tables 2, 3).

Sensitivity analyses using ITT population with multiple imputation showed similar results in primary and secondary outcomes (see Supplementary Tables 1, 2).

### Cost-effectiveness outcomes

The average total cost per patient over 12 weeks for the TELE group was significantly lower than for the FTF group. The total cost for the TELE group was 58,303.18 CNY compared to 82,358.90 CNY for the FTF group, with a *p*-value of less than 0.001, indicating a significant difference. The cost of physical therapy was a major contributor, with the TELE group incurring 6980.00 CNY while the FTF group incurred 23,308.62 CNY, also with a significant *p*-value (< 0.001). Hospital stay costs were similar between the two groups (*p* = 0.903). Other medical costs, such as primary care, were substantially lower in the TELE group (163.64 CNY vs. 2,118.97 CNY, *p* < 0.001). However, non-medical costs, such as transportation, were also lower in the TELE group (711.52 CNY vs. 1,125.83 CNY, *p* = 0.009). Opportunity costs, including lost wages, were higher for the TELE group, although the differences were not statistically significant (Table 4).

**Table 4:** Average total cost per patient in the TELE and FTF groups during the 12 weeks after the surgery

Cost category (CNY)	TELE group (N = 33)	FTF group (N = 29)	*P* value
Intervention cost			
Physical therapist cost	6980.00 (0.00)	23,308.62 (1790.75)	< 0.001
Hospital stay cost	5419.42 (1240.02)	5381.69 (1187.83)	0.903
Other medical cost			
Primary care	163.64 (50.42)	2118.97 (162.80)	< 0.001
Secondary care	1133.70 (1017.21)	1548.31 (1480.74)	0.199
Paid home care	192.70 (196.22)	294.83 (290.16)	0.106
Medication	5216.06 (1074.05)	5685.59 (1547.77)	0.166
Non-medical cost			
Transportation cost	711.52 (557.00)	1125.83 (659.92)	0.009
Nutrition cost	1943.64 (660.84)	1805.35 (619.35)	0.401
Opportunity cost			
Lost wages for patients	33,459.79 (17,910.55)	39,147.66 (20,426.36)	0.247
Lost wages for families	3082.73 (3598.28)	1942.07 (3249.20)	0.198
Total cost	58,303.18 (18,408.65)	82,358.90 (21,342.71)	< 0.001

TELE, telerehabalitation; FTF, face-to-face rehabilitation; CNY, Chinese Yuan

The ICER analysis indicated that the TELE group was more cost-effective than the FTF group. At 12 weeks, the incremental cost for the TELE group was − 24,055.71 CNY, meaning a cost saving compared to the FTF group. However, the incremental effectiveness in terms of IKDC, LEFS, SF-12 PCS, SF-12 MCS, and active ROM was minimal and non-significant. The ICER for the IKDC score was − 158,767.64 CNY per point gained, indicating substantial cost savings per unit of effectiveness, but with no significant clinical difference. Other ICER values, such as for the LEFS, SF-12 PCS, and active ROM, also reflected cost savings, though the incremental improvements were small and not statistically significant (Table 5).

**Table 5:** Incremental cost-effectiveness ratio (ICER)

	Incremental cost, CNY	Incremental IKDC score	Incremental LEFS score	Incremental SF-12 PCS	Incremental SF-12 MCS	Incremental active ROM (°)	ICER (IKDC score)	ICER (LEFS score)	ICER (SF-12 PCS)	ICER (SF-12 MCS)	ICER (Active ROM)
Per protocol, mixed effects, week 12	− 24,055.71 (− 34,152.90, − 13,958.53)	0.15 (− 5.81, 6.11)	− 1.71 (− 6.99, 3.57)	− 2.40 (− 6.24, 1.44)	− 0.06 (− 5.60, 5.47)	2.83 (− 5.12, 10.78)	− 158,767.64	14,063.11	10,033.22	394,520.18	− 8,498.09
ITT using multiple imputation, mixed effects, week 12	− 24,055.71(− 34,152.90, − 13,958.53)	0.46 (− 5.50, 6.43)	− 2.78 (− 7.99, 2.44)	− 2.72 (− 6.33, 0.90)	1.10 (− 4.25, 6.45)	2.83 (− 5.12, 10.78)	− 51,736.59	8664.15	8859.65	− 21,861.61	− 8,498.09

CNY, Chinese Yuan; IKD, International Knee Documentation Committee Subjective Knee Form; LEFS, lower extremity functional scale; SF-12, 12-item short form survey; PCS, physical component score; MCS, mental component score; ROM, range of motion

### Adherence and acceptability

Adherence rates were high, the TELE group averaged 5.2 sessions per week, while the FTF group averaged 5.1 sessions. Additionally, participants in both groups reported positive perceptions of the treatment received, with average scores of 8.7 out of 10 for all evaluated aspects, as documented in Table 6. There was no significant difference between groups with regard to participant-reported satisfaction (Table 6).

**Table 6:** Patients’ adherence to treatment

Outcome measure	TELE group (N = 33)	FTF group (N = 29)	*P* value
Number of sessions performed per week, mean ± SD	5.2 ± 1.1	5.1 ± 1.0	0.554
Agreement with the following questions (0 to 10)*, mean (SD)			
To what extent did you agree to accept the allocated exercise plan?	8.3 ± 1.3	8.6 ± 1.2	0.094
To what extent did you do the exercise program as recommended?	8.6 ± 1.4	8.7 ± 1.4	0.727
To what extent do you agree that the intervention relieved your pain?	8.7 ± 1.4	8.8 ± 1.3	0.933
To what extent do you agree that the intervention improved your function?	8.9 ± 1.2	9.0 ± 1.1	0.802
To what extent were you satisfied with the exercise protocol?	9.5 ± 0.5	9.6 ± 0.5	0.805

N/A, not applicable

*0 = strongly disagree, 10 = strongly agree

### Adverse events

Throughout the follow-up period, the incidence of adverse events was comparable between the two groups. No serious events were linked to the telerehabilitation intervention, although one minor event was potentially associated with the FTF intervention (Table 7). The rates of participants lost to follow-up were also similar across both groups, with the majority of losses happening during the final follow-up survey (Fig. 1).

**Table 7:** Adverse events and serious adverse events

Adverse events	TELE group (N = 33)	PT group (N = 29)
Patients with adverse events (no. [%])	5 (15.2)	6 (20.7)
Events related to study therapy (no.)	0	1
Events unrelated to study therapy (no.)	7	7
Type of event (no.)		
Involved knee		
Pain	3	2
Swelling	2	2
Muscle strain	0	1#
Signs of infection (swelling, redness, heat, or pus)	0	
Mobilization under anesthesia	0	
Other		
Nausea and dizziness	0	1
Back pain	0	1
Anxiety about knee recovery	2	1
Serious adverse events*		
Patients with serious adverse events (no. [%])	0	0
Events related to study therapy (no.)	0	0
Events unrelated to study therapy (no.)	0	0

^#^One patient sustained gastrocnemius muscle strain during intervention with minor consequent symptoms

*Patients with serious adverse events were automatically withdrawn from the study

## Discussion

This is the first study to examine the clinical and cost-effectiveness of telerehabilitation compared with hospital/clinic-based face-to-face rehabilitation for patients after arthroscopic ACL reconstruction in China. In the context of ACL injury care, factors such as delayed surgery and debates on optimal surgical timing have been examined [24, 25]. Recent literature has also explored various facets of ACL reconstruction and rehabilitation, including lateral extra-articular surgical procedures, novel graft choices (e.g., patellar tendon autografts), and evolving rehabilitation protocols [26–28]. The mean treatment effect of participants was assessed at week 6 and week 12 postoperatively. A significant improvement was found within both the TELE and FTF groups from baseline to week 6 and week 12. Specifically, IKDC scores improved in both groups, with no significant difference between them. As an important knee kinematics score, the IKDC reflects recovery of knee function after arthroscopic ACL reconstruction [29]. Similarly, emphasis on postural control has been linked to successful return to sport after ACL reconstruction [30]. Therefore, these findings indicate that telerehabilitation training can, like in-person physiotherapy, effectively restore joint function. This is supported by evidence that telerehabilitation can effectively reduce pain and disability in musculoskeletal conditions [31]. Structured periodization has also been recommended to optimize ACL rehabilitation [32]. A systematic review and meta-analysis found that home-based telerehabilitation programs achieve comparable long-term outcomes in pain, mobility, physical function, and patient-reported health status to hospital-based programs after primary total knee arthroplasty [33]. Zhao et al. also reported no significant differences in knee flexion and extension between home-based and outpatient rehabilitation after knee arthroplasty [34]. Notwithstanding these encouraging early outcomes, ACL reconstruction entails a protracted rehabilitation trajectory, often 6–12 months to meet return-to-sport criteria, so our 12-week data reflect early-phase recovery and cannot, on their own, establish long-term clinical equivalence [3, 28, 32].

LEFS is designed to evaluate the functional abilities of patients with lower extremity disabilities by measuring the difficulty they experience in completing daily activities [35, 36]. In ACL patients, deficits in knee proprioception and quadriceps force control are common, highlighting the importance of comprehensive functional assessment [37, 38]. In this study, knee function improved in both groups with no significant differences between them. Knee joint range of motion (ROM) is a key factor in postoperative recovery after arthroscopic ACL reconstruction, and we found no significant between-group differences in ROM (flexion and extension), except at week 12, where the FTF group had a slightly higher mean flexion improvement than TELE, although this difference was not statistically significant. Aquatic rehabilitation programs have been shown to yield comparable knee flexion outcomes after ACL reconstruction [39]. Nevertheless, the majority of patients in both groups achieved knee flexion of 120° or more, with no significant differences in the proportions reaching this standard flexion range. This is consistent with evidence that structured neuromuscular training can improve functional classification and that concurrent meniscal repair does not impair isokinetic recovery after ACL reconstruction [40, 41]. Indeed, isolated ACL deficiency primarily impacts knee stability rather than altering standard functional score [42]. Thus, the analysis suggests that the TELE group is also effective in helping patients reach the 120° knee flexion goal by phase three of rehabilitation.

Previous research has indicated that mental health can deteriorate in patients with ACL injuries [43]. ACL tears may induce neuroplastic changes and psychological stress, so addressing emotional health is important [44, 45]. In this study, both groups showed substantial improvements in negative emotions related to ACL injuries and postoperative pain, as reflected in their improved SF-12 scores. By week 12, significant gains were observed in the MCS-12 and PCS-12 scores of both groups, though some PCS-12 scores remained above 50. Despite physiotherapist guidance, fully restoring pre-injury knee function by week 12 was challenging for many, which may have contributed to the lower PCS-12 scores.

In line with a study in Norway on patients with musculoskeletal problems, this study indicated that telerehabilitation therapy was cost-saving [46]. Both cost and health benefits of the interventions could influence cost-effectiveness. Our analysis showed that telerehabilitation was less costly than face-to-face intervention. A cost analysis in Canada on patients with a knee problem also concluded that the cost of telerehabilitation was lower than that of conventional rehabilitation [47]. Other studies in knee arthroplasty rehabilitation indicate the effectiveness of home-based programs. For instance, home exercise programs were found not inferior to outpatient physical therapy in recovery of knee flexion after total knee arthroplasty [48]. A scoping review highlighted the potential of telerehabilitation to change the standard of care, particularly in low- to middle-income countries with scarce healthcare provider distribution [49].

The increment or reduction of the costs and effectiveness of the telerehabilitation by half from the base case values was unlikely to affect its cost-effectiveness in this study. The findings of this study are consistent with the results of the cost-effectiveness analysis study on telemedicine for primary care delivery, where telemedicine was shown to be cost saving as long as its effectiveness was greater than that of the controlled intervention [50]. However, the reduction of the health benefits from the base case values in this study could lead telerehabilitation not to be a cost-effective intervention. Overall, it is important that patients adhere to telerehabilitation services and improve their health for the new intervention to be cost-effective.

The TELE intervention cost approximately 50% less than FTF, largely due to fewer facility requirements and reduced therapist time. In other words, there is an opportunity to implement telerehabilitation programs across numerous geographic locations if needed. In middle-income countries, such as China, access to physiotherapy services is a challenge because of the shortage of physiotherapists and limited access to hospital/clinic -based programs [51]. Unlike FTF, TELE could overcome barriers to accessing physiotherapy services and could provide numerous benefits with reduced cost to the patients in China. However, the key challenges for its key barriers include the availability of reliable internet service and patient reluctance to engage [52]. Moreover, patient digital literacy, internet connectivity, and motivation could influence adherence to telerehabilitation. Lack of broadband access and low e-health literacy, for instance, have been shown to limit engagement with remote health programs. Highly motivated patients might also adhere more closely regardless of format. Because we did not directly assess these factors, they may bias our adherence estimates. We have added this caveat here to acknowledge these potential limitations.

The major strength of this study was that it is the first study in low- and middle-income countries to evaluate the cost-effectiveness of telerehabilitation therapy for patients after ACL reconstruction using a randomized controlled trial. In addition, the findings of this study could inform clinicians and decision makers about the implementation of TELE as a complementary option of FTF services in China. On the other hand, the findings reported here should be viewed in the context of the limitations of this study. One such limitation is that the trial was open-label (only outcome assessors were blinded), which could introduce performance bias, especially given that our primary and secondary outcomes (IKDC, LEFS, NPRS, SF-12) are subjective patient-reported measures that may be influenced by patients’ expectations. In addition, we did not directly measure quadriceps or hamstring strength, nor did we include validated functional performance tests (such as single-leg hop tests), which limits our ability to evaluate neuromuscular recovery and return-to-sport readiness. The second limitation of the study was related to the time of follow-up; the effects of the telerehabilitation therapies might be different in the long-term follow-up. Specifically, comprehensive recovery after ACL reconstruction commonly spans 6–12 months; therefore, endpoints such as return-to-sport readiness, re-injury/graft failure rates, persistent symptoms, objective strength/proprioception, and sustained cost profiles should be assessed over this longer horizon to verify TELE–FTF equivalence. Thus, evidence of health benefits from a long-term follow-up of patients is important to be incorporated in the cost-effectiveness analysis of telerehabilitation. In addition, our study was conducted at a single trauma center in Shanghai with a relatively small sample size (N = 68), which may limit the generalizability of our findings and increase the risk of selection bias.

In conclusion, the findings of this study showed that telerehabilitation was associated with comparable health benefits and lower costs, suggesting that it was a cost-saving therapy compared with a hospital/clinic -based face-to-face rehabilitation. Because ACL rehabilitation usually requires 6–12 months to achieve full functional recovery, these conclusions pertain to short-term outcomes only, and longer-term follow-up is necessary to confirm clinical equivalence. This suggests that the implementation of telerehabilitation could help to overcome barriers to access to physiotherapy services, particularly in middle-income countries such as China, thereby improving the health outcomes of patients in these countries. Future studies are required to assess the cost-effectiveness of the intervention in the long term from the patient and societal perspective.

## Clinical messages

### Findings

The study found that at the 12-week follow-up, there were no significant differences between telerehabilitation (TELE) and face-to-face rehabilitation (FTF) groups in terms of knee function, pain, or range of motion (ROM). The mean differences for various measurements (IKDC, LEFS, SF-12, NPRS, ROM) were close to zero. The average cost per person was significantly lower for the TELE group (58,303.18 CNY) compared to the FTF group (82,358.90 CNY), with the TELE intervention being more cost-effective.

### Implications

This study suggests that telerehabilitation is non-inferior to traditional face-to-face rehabilitation for patients post-ACL reconstruction, providing similar clinical outcomes at a lower cost. The results suggest that telerehabilitation could be a viable alternative in settings where access to in-person rehabilitation is limited, potentially impacting clinical practice by offering a more accessible and cost-effective approach to rehabilitation.

### Limitations

A key limitation of this study is its external validity; the results may not be generalizable to populations outside Shanghai, as the study was conducted at a single trauma center. The specific socio-economic and healthcare context in Shanghai may limit the applicability of these findings to other regions or healthcare systems. Additionally, the relatively small sample size (n = 68) may reduce statistical power and increase the risk of selection bias, further limiting the robustness and generalizability of the findings. Moreover, all participants in our study had access to smartphones and home Wi-Fi, which may not reflect the broader patient population. In many settings, unequal access to reliable internet and devices, often termed the “digital divide”, can significantly hinder telehealth uptake. Older or less tech-savvy individuals may also perceive telemedicine as complex due to limited technical skills or confidence. Together, these factors could limit the generalizability of our findings and dampen the adoption of telerehabilitation, and should be considered when interpreting results.

## Supplementary Information

Below is the link to the electronic supplementary material.

Supplementary file1 (DOC 38 kb)

Supplementary file2 (DOC 45 kb)

Supplementary file3 (DOC 45 kb)

## Data Availability

Data is available upon request (contact the corresponding author after online publication). All participants’ baseline characteristics as well as data obtained during follow-up period are available in blind format.

## References

[1] Mall NA, Chalmers PN, Moric M, et al. Incidence and trends of anterior cruciate ligament reconstruction in the United States. Am J Sports Med. 2014;42:2363–70. 25086064 10.1177/0363546514542796

[2] Kaeding CC, Leger-St-Jean B, Magnussen RA. Epidemiology and diagnosis of anterior cruciate ligament injuries. Clin Sports Med. 2017;36:1–8. 27871652 10.1016/j.csm.2016.08.001

[3] Musahl V, Karlsson J. Anterior cruciate ligament tear. N Engl J Med. 2019;380:2341–8. 31189037 10.1056/NEJMcp1805931

[4] Anderson AF, Snyder RB, Lipscomb AB Jr. Anterior cruciate ligament reconstruction. a prospective randomized study of three surgical methods. Am J Sports Med. 2001;29:272–9. 11394593 10.1177/03635465010290030201

[5] Sanders TL, Pareek A, Hewett TE, et al. Long-term rate of graft failure after ACL reconstruction: a geographic population cohort analysis. Knee Surg Sports Traumatol Arthrosc: Of J ESSKA. 2017;25:222–8. 10.1007/s00167-016-4275-y 27522592

[6] Tedeschi R, Giorgi F. What is known about the RegentK regenerative treatment for ruptured anterior cruciate ligament? A scoping review. Man Med. 2023;61:181–7.

[7] Azma K, RezaSoltani Z, Rezaeimoghaddam F, Dadarkhah A, Mohsenolhosseini S. Efficacy of tele-rehabilitation compared with office-based physical therapy in patients with knee osteoarthritis: a randomized clinical trial. J Telemed Telecare. 2018;24(8):560–5. 28771070 10.1177/1357633X17723368

[8] Galea MD. Telemedicine in rehabilitation. Phys Med Rehabil Clin N Am. 2019;30(2):473–83. 30954160 10.1016/j.pmr.2018.12.002

[9] Bailey JF, Agarwal V, Zheng P, Smuck M, Fredericson M, Kennedy DJ, et al. Digital care for chronic musculoskeletal pain: 10,000 participant longitudinal cohort study. J Med Internet Res. 2020;22(5):e18250. 32208358 10.2196/18250 PMC7248800

[10] Tedeschi R, Platano D, Pillastrini P, Berti L, Benedetti MG. Effectiveness of tele-rehabilitation in patients with knee osteoarthritis: a randomized controlled trial. Digit Health. 2024;10:20552076241286184. 39493627 10.1177/20552076241286186 PMC11528740

[11] Lin CC, Haas M, Maher CG, Machado LA, van Tulder MW. Cost-effectiveness of guideline-endorsed treatments for low back pain: a systematic review. Eur Spine J. 2011;20(7):1024–38. 21229367 10.1007/s00586-010-1676-3 PMC3176706

[12] Oliveira VC, Ferreira PH, Maher CG, Pinto RZ, Refshauge KM, Ferreira ML. Effectiveness of self-management of low back pain: systematic review with meta-analysis. Arthritis Care Res (Hoboken). 2012;64(11):1739–48. 22623349 10.1002/acr.21737

[13] Irrgang JJ, Anderson AF, Boland AL, et al. Development and validation of the International knee documentation committee subjective knee form. Am J Sports Med. 2001;29:600–13. 11573919 10.1177/03635465010290051301

[14] Crawford K, Briggs KK, Rodkey WG, et al. Reliability, validity, and responsiveness of the IKDC score for meniscus injuries of the knee. Arthrosc: J Arthrosc Relat Surg. 2007;23:839–44. 10.1016/j.arthro.2007.02.005 17681205

[15] van de Graaf VA, Wolterbeek N, Scholtes VA, et al. Reliability and validity of the IKDC, KOOS, and WOMAC for patients with meniscal injuries. Am J Sports Med. 2014;42(6):1408–16. 24618098 10.1177/0363546514524698

[16] Jenkinson C, Layte R. Development and testing of the UK SF-12 (short form health survey). J Health Serv Res Policy. 1997;2(1):14–8. 10180648 10.1177/135581969700200105

[17] Ware J Jr., Kosinski M, Keller SD. A 12-item short-form health survey: construction of scales and preliminary tests of reliability and validity. Med Care. 1996;34(3):220–33. 8628042 10.1097/00005650-199603000-00003

[18] Burdine JN, Felix MR, Abel AL, et al. The SF-12 as a population health measure: an exploratory examination of potential for application. Health Serv Res. 2000;35(4):885–904. 11055454 PMC1089158

[19] Gill SC, Butterworth P, Rodgers B, et al. Validity of the mental health component scale of the 12-item short-form health survey (MCS-12) as measure of common mental disorders in the general population. Psychiatry Res. 2007;152(1):63–71. 17395272 10.1016/j.psychres.2006.11.005

[20] Ware JE, Kosinski MA, Keller SD. SF-12: How to score the SF-12 physical and mental health summary scales. 2002.

[21] Bouwmans C, Krol M, Severens H, et al. The iMTA productivity cost questionnaire: a standardized instrument for measuring and valuing health-related productivity losses. Value Health. 2015;18:753–8. 26409601 10.1016/j.jval.2015.05.009

[22] Circular of the Shanghai Municipal Bureau of human resources and social security on the average wages of employed persons in full-caliber urban units in the city in 2022. Available: https://rsj.sh.gov.cn/.

[23] Hinman RS, Lawford BJ, Campbell PK, Briggs AM, Gale J, Bills C, et al. Telephone-delivered exercise advice and behavior change support by physical therapists for people with knee osteoarthritis: protocol for the telecare randomized controlled trial. Phys Ther. 2017;97:524–36. 28339847 10.1093/ptj/pzx021

[24] Giordano L, Maffulli N, Carimati G, et al. Increased time to surgery after anterior cruciate ligament tear in female patients results in greater risk of medial meniscus tear: a study of 489 female patients. Arthroscopy. 2023;39(3):613–22. 36309227 10.1016/j.arthro.2022.10.014

[25] Maffulli N. The early versus late anterior cruciate ligament reconstruction debate: history teaches us that we cannot use reason and evidence to fight and win against conviction. Arthroscopy. 2018;34(9):2524–5. 30173789 10.1016/j.arthro.2018.06.017

[26] Migliorini F, Lucenti L, Mok YR, et al. Anterior cruciate ligament reconstruction using lateral extra-articular procedures: a systematic review. Medicina (Kaunas). 2025;61(2):294. 40005410 10.3390/medicina61020294 PMC11857574

[27] Agrawal S, Hegde AS, Rao BS, et al. Peroneus longus tendon autograft for primary arthroscopic reconstruction of the anterior cruciate ligament. Muscles Ligaments Tendons J. 2023;13(2):252–8.

[28] Piedade SR, Leite Arruda BP, de Vasconcelos RA, et al. Rehabilitation following surgical reconstruction for anterior cruciate ligament insufficiency: what has changed since the 1960s? State of the art. J ISAKOS. 2023;8(3):153–62. 36410671 10.1016/j.jisako.2022.10.001

[29] Rossi MJ, Lubowitz JH, Guttmann D. Development and validation of the International Knee Documentation Committee subjective knee form. Am J Sports Med. 2002;30(1):152. 11799013 10.1177/03635465020300011301

[30] Papalia R, Franceschi F, Te Cambe A, et al. Anterior cruciate ligament reconstruction and return to sport activity: postural control as the key to success. Int Orthop. 2015;39(3):527–34. 25192689 10.1007/s00264-014-2513-9

[31] Kosterink SM, Huisin’t Veld RM, Cagnie B, et al. The clinical effectiveness of a myofeedback-based teletreatment service in patients with nonspecific neck and shoulder pain: a randomized controlled trial. J Telemed Telecare. 2010;16(6):316–21. 20798425 10.1258/jtt.2010.006005

[32] Kakavas G, Malliaropoulos N, Bikos G, et al. Periodization in anterior cruciate ligament rehabilitation: a novel framework. Med Princ Pract. 2021;30(2):101–8. 33264774 10.1159/000511228 PMC8114043

[33] Zhang H, Wang J, Jiang Z, et al. Home-based tele-rehabilitation versus hospital-based outpatient rehabilitation for pain and function after initial total knee arthroplasty: a systematic review and meta-analysis. Medicine (Baltimore). 2023;102(51):e36764. 38134064 10.1097/MD.0000000000036764 PMC10735162

[34] Zhao B, Liu H, Du K, et al. Effectiveness and safety of outpatient rehabilitation versus home-based rehabilitation after knee arthroplasty: a systematic review and meta-analysis. J Orthop Surg Res. 2023;18(1):704. 37726800 10.1186/s13018-023-04160-2 PMC10510230

[35] Yeung TS, Wessel J, Stratford P, et al. Reliability, validity, and responsiveness of the Lower Extremity Functional Scale for inpatients of an orthopaedic rehabilitation ward. J Orthop Sports Phys Ther. 2009;39(6):468–77. 19487822 10.2519/jospt.2009.2971

[36] Binkley JM, Stratford PW, Lott SA, et al. The lower extremity functional scale (LEFS): scale development, measurement properties, and clinical application. Phys Ther. 1999;79(4):371–83. 10201543

[37] Jebreen M, Maffulli N, Migliorini F, et al. Known-group validity of passive knee joint position sense: a comparison between individuals with unilateral anterior cruciate ligament reconstruction and healthy controls. J Orthop Surg Res. 2023;18(1):525. 37481595 10.1186/s13018-023-03996-y PMC10363318

[38] Lemos T, Albarello JCS, Silva SC, Mozella AP. Quadriceps force fluctuation during maximal isometric contraction is altered in ACL injury and is associated with lower limb functional performance: a cross-sectional study with athletes. Muscles Ligaments Tendons J. 2024;14(3):499–506.

[39] Pipino G, Tomasi E, Mardones R, et al. Rehabilitation after anterior cruciate ligament reconstruction: dry land vs aquatic rehabilitation. Muscles Ligaments Tendons J. 2023;13(3):421–9.

[40] Maffulli N, Oliva F. Coper classification early after ACL rupture changes with progressive neuromuscular and strength training and is associated with 2-year success: letter to the editor. Am J Sports Med. 2019;47(11):NP64–5. 31479329 10.1177/0363546519863310

[41] Albarello JCS, Laett CT, Soares de Palma AM, et al. Associated ACL reconstruction and meniscal repair do not affect the evolution of isokinetic parameters in professional athletes: a prospective study with a one-year follow-up. Muscles Ligaments Tendons J. 2024;14(3):450–7.

[42] Ramjug S, Ghosh S, Walley G, Maffulli N. Isolated anterior cruciate ligament deficiency, knee scores and function. Acta Orthop Belg. 2008;74(5):643–51. 19058699

[43] Hong CK, Liu ZW, Hsu KL, et al. A novel home-based rehabilitative knee brace system is a viable option for postoperative rehabilitation after anterior cruciate ligament reconstruction: a report of 15 cases. J Exp Orthop. 2022;9(1):96. 36149519 10.1186/s40634-022-00538-z PMC9508297

[44] Kakavas G, Malliaropoulos N, Pruna R, et al. Neuroplasticity and anterior cruciate ligament injury. Indian J Orthop. 2020;54(3):275–80. 32399146 10.1007/s43465-020-00045-2 PMC7205971

[45] Maffulli N, King JB. Anterior cruciate ligament injury. Br J Sports Med. 1998;32(3):266. 9773185

[46] Buvik A, Bergmo TS, Bugge E, et al. Cost-effectiveness of telemedicine in remote orthopedic consultations: randomized controlled trial. J Med Internet Res. 2019;21(2):e11330. 30777845 10.2196/11330 PMC6399572

[47] Tousignant M, Moffet H, Boissy P, et al. Cost analysis of in-home telerehabilitation for post-knee arthroplasty. J Med Internet Res. 2015;17(2):e38. 25840501 10.2196/jmir.3844 PMC4397389

[48] Browne JA, Bedard NA, Buchanan MR, et al. After unilateral total knee arthroplasty, unsupervised home exercise programs were noninferior to outpatient physiotherapy for increasing passive knee flexion: a prospective cohort study. J Bone Joint Surg Am. 2019;101(21):2063–73. 31764371 10.2106/JBJS.19.00987

[49] Cottrell MA, Galea OA, O’Leary SP, et al. Real-time telerehabilitation for the treatment of musculoskeletal conditions is effective and comparable to standard practice: a systematic review and meta-analysis. Phys Ther Rev. 2017;22(5–6):1–24. 27141087 10.1177/0269215516645148

[50] Li Q, et al. Tele-rehabilitation in developing countries: a review. J Telemed Telecare. 2021;27(9):529–37.

[51] Wong KL, et al. Patient acceptance of mobile health technology for rehabilitation. Telemed J E Health. 2020;26(4):543–51.

[52] Maffulli N, Osti L. ACL stability, function, and arthritis: what have we been missing? Orthopedics. 2013;36(2):902. 10.3928/01477447-20130122-02 23379658

