# Supplementary material for: The efficacy and cost-effectiveness analysis of telerehabilitation for patients after arthroscopic ACL-reconstruction: a non-inferiority randomized controlled trial
Source: J Orthop Surg Res. 2025 Dec 24;20:1085. doi: 10.1186/s13018-025-06403-w (PMC12729216; doi:10.1186/s13018-025-06403-w)
Supplement: Supplementary file 1 — Supplementary file1 (DOC 38 kb) [file 13018_2025_6403_MOESM1_ESM.doc]

**Supplemental Materials**

**Materials and Methods**

**Intervention**

Standard Postoperative Rehabilitation Program for Arthroscopic ACLR Surgery in the Early Stage.

Stage1: After surgery - 2 weeks after surgery

1. Ankle pump: Lie flat on back with legs straight. Dorsiflexion and extension of the ankles to maximum range of motion, alternating feet. (Repeat 15 times. Do 3 sessions per day)
2. Quadriceps contraction exercise: Lie flat on back with legs straight. Put a towel roll under the knee. Contract the quadriceps to press the towel roll below the knee and hold for 5seconds. (Repeat 10 times. Do 3 sessions per day)
3. Straight leg raise exercise: Lie flat on back with legs straight. Point your toes to the ceiling. Quadriceps contract and straight knee raises. Lift your leg to 60° and hold for 3 seconds. (Repeat 10 times. Do 3 sessions per day)
4. Patella movement: Straighten the knee and push the patella in all 4 directions with the fingers stuck on the patella to the maximum range for 2 seconds. (Repeat 10 times. Do 3 sessions per day)
5. Knee straightening exercise: Straighten the knee and pad the foam shaft under the ankle. Press the hands above the knee gently to increase the pulling sensation on the back of the thigh for 15-20 seconds. (5 minutes at a time. 3-5 times per day)
6. Weight-bearing exercise in the standing position: Stand with the knee brace on, keep the knee straight, and hold the back of the chair with both hands. Shift the center of gravity to the affected side for 3 seconds and then shift it to the healthy side. (Repeat 10 times. Do 3 sessions per day)
7. Flexion exercise in lying position: With the legs straight and lying flat on the bed, the heel of the affected side is pushed along the bed towards the buttocks to the maximum tolerated flexion angle and hold for 2 seconds before returning to the starting position. (This maneuver can be performed with the help of another person. Repeat 10 times. Do 3 sessions per day)
8. Hip extension exercise in prone position: Lie face down on the bed with both legs straight. Lift the affected leg off the bed and feel the gluteal muscles contract and activate. (This maneuver can be performed with the help of another person. Repeat 10 times. Do 3 sessions per day)
9. Walk with crutches: Extend the crutches first, and then take the next step with the affected limb. Then move the center of gravity forward and step out with healthy lower limb. (5 minutes at a time. 3 times per day)

Stage2: 3 weeks after surgery- 4 weeks after surgery

Add the following moves to previous workouts.

1. Scar relaxation: Use deep, slow force with hands to push the tissue around the scar. (1 minute at a time. 3 times per day)
2. Flexion exercise in sitting position: Sit with feet touching the floor and slide the affected limb backward along the floor to the end. Use the healthy calf to press on the affected leg to assist in bending the knee to the maximum angle and hold for 5 seconds. (Repeat 10 times. Do 3 sessions per day)
3. 0-30° shallow squat exercise：Wearing the brace, hold support level with both hands and lean forward. Squat down and bend knees 30°. (Repeat 10 times. Do 3 sessions per day)
4. Forward strides with weight shifting: Stand with the brace on. Step forward with the affected limb and shift the center of gravity forward. Then, step forward with the healthy limb and stand firmly. Withdraw the center of gravity successively, starting with the healthy side moving backward first, followed by the affected limb moving back to the starting position. (Repeat 10 times. Do 3 sessions per day)
5. Lateral strides with weight shifting: Stand with the brace on. Step the affected limb to the side, shift the center of gravity to the affected limb, then step the healthy limb forward, walk laterally for 2-3 steps, and then move in the opposite direction. (Repeat 10 times. Do 3 sessions per day)
6. Single leg standing exercise: Stand with the brace on, shift your weight to the affected limb, and lift the healthy side off the ground for 30 seconds. (1 minute at a time. 3 times per day)
7. Unilateral crutch walking exercise (substitute exercise 9): The crutch is placed on the healthy side, the crutch is extended forwards first, the affected side then steps forwards and finally the healthy side follows. (1 minute at a time. 3 times per day)

Stage3: 5 weeks after surgery- 6 weeks after surgery

Add the following moves to previous workouts.

1. Weighted straight leg raise exercise: Do exercise 3 with sandbags place around the ankles. (Repeat 10 times. Do 3 sessions per day)
2. Hamstring exercise in the prone position: Lie face down on the bed with an elastic band tied around one ankle and the other end secured to the foot of the bed or held in place by a partner. Then, hook calf back and hold the end of the band for 3 seconds. Avoid raising pelvis during the exercise. (Repeat 10 times. Do 3 sessions per day)
3. Squat against wall: Half a meter from the wall, squat with your back against the wall to 120°. (30 seconds at a time. 3-5 times per day)
4. Heel lifting exercise: Stand on both feet and apply force to the forefoot. Lift the heel to the maximum angle and then slowly lower it. Keep the knee straight during the exercise. As the exercise progresses, gradually transition to standing on the affected limb on one foot. (Repeat 10 times. Do 3 sessions per day)

Stage4: 7 weeks after surgery- 12 weeks after surgery

Add the following moves to previous workouts.

1. Unipedal balance exercise: Stand on one foot on a balance mat with the affected limb and hold for 30 seconds. (Repeat 10 times. Do 3 sessions per day)
2. Walking exercise: Do exercise 16 without crutches and brace. As the exercise progresses, gradually accelerate walking speed. (1 minute at a time. 3 times per day)
3. Stationary bicycle riding exercise: Ride a stationary bicycle for 30 minutes. (3 times per day)
4. Alternate up and down steps: To go up and down steps, alternate between using the affected limb and the healthy side. (Repeat 10 times. Do 3 sessions per day)
5. Quadratic skipping: Feet together with arms akimbo, jump back and forth and side to side 10 times in a row. (Repeat 10 times. Do 3 sessions per day)
6. 0-120° squat exercise: Do exercise 12 without brace and squat to maximum range of motion. (Repeat 10 times. Do 3 sessions per day)
