# Supplementary material for: The efficacy and cost-effectiveness analysis of telerehabilitation for patients after arthroscopic ACL-reconstruction: a non-inferiority randomized controlled trial
Source: J Orthop Surg Res. 2025 Dec 24;20:1085. doi: 10.1186/s13018-025-06403-w (PMC12729216; doi:10.1186/s13018-025-06403-w)
Supplement: Supplementary file 2 — Supplementary file2 (DOC 45 kb) [file 13018_2025_6403_MOESM2_ESM.doc]

Table S1 Changes in outcomes for the TELE and FTF groups at weeks 6 and 12 after surgery, ITT analysis using multiple imputation

| Outcome | 6 weeks post surgery | | |  | 12 weeks post surgery | | |
| --- | --- | --- | --- | --- | --- | --- | --- |
|  | TELE group (N=35) | FTF group (N=33) | P value |  | TELE group (N=35) | FTF group (N=33) | P value |
| IKDC | -5.62 (12.65) | -10.56 (15.42) | 0.153 |  | 7.37 (13.20) | 6.91 (14.16) | 0.889 |
| IKDC (MAX adjusted to 100) | -6.47 (14.54) | -12.14 (17.72) | 0.153 |  | 8.47 (15.18) | 7.94 (16.27) | 0.889 |
| LEFS | -4.75 (10.94) | -3.32 (13.87) | 0.638 |  | 8.11 (11.59) | 10.89 (12.55) | 0.346 |
| SF-12 PCS | -1.65 (7.73) | 1.29 (7.45) | 0.115 |  | 3.21 (8.78) | 5.92 (8.47) | 0.199 |
| SF-12 MCS | -0.18 (9.40) | -3.99 (15.10) | 0.213 |  | 2.83 (9.77) | 1.73 (13.10) | 0.695 |
| NPRS | -0.14 (1.86) | -0.35 (1.81) | 0.633 |  | -0.84 (1.61) | -1.14 (1.49) | 0.443 |
| Active Knee Flexion (°) | -11.75 (15.58) | -17.18 (18.19) | 0.190 |  | 2.19 (11.75) | -0.22 (12.41) | 0.414 |
| Active Knee Extension (°) | 2.73 (5.88) | 3.97 (5.57) | 0.400 |  | -0.15 (3.18) | -0.17 (3.89) | 0.982 |
| Active ROM (°) | -14.18 (19.24) | -20.10 (21.02) | 0.251 |  | 2.73 (12.63) | -0.10 (14.78) | 0.419 |
| Passive Knee Flexion (°) | -13.85 (17.57) | -17.86 (17.70) | 0.375 |  | 2.00 (13.76) | -0.79 (13.20) | 0.420 |
| Passive Knee Extension (°) | 1.97 (5.44) | 2.93 (5.59) | 0.496 |  | 0.15 (4.59) | 0.34 (5.50) | 0.881 |
| Passive ROM (°) | -15.82 (19.28) | -20.79 (21.01) | 0.335 |  | 1.85 (15.31) | -1.14 (16.60) | 0.464 |
| TELE = telerehabalitation; FTF = face-to-face rehabilitation; IKDC= International Knee Documentation Committee Subjective Knee Form; LEFS: Lower Extremity Functional Scale; SF-12=12-item Short Form Survey; PCS= Physical Component Score; MCS= Mental Component Score; NPRS= Numeric Pain Rating Scale; ROM= Range of Motion. | | | | | | | |
