# Supplementary material for: The efficacy and cost-effectiveness analysis of telerehabilitation for patients after arthroscopic ACL-reconstruction: a non-inferiority randomized controlled trial
Source: J Orthop Surg Res. 2025 Dec 24;20:1085. doi: 10.1186/s13018-025-06403-w (PMC12729216; doi:10.1186/s13018-025-06403-w)
Supplement: Supplementary file 3 — Supplementary file3 (DOC 45 kb) [file 13018_2025_6403_MOESM3_ESM.doc]

Table S2 Effectiveness estimates from linear mixed effects models, ITT analysis using multiple imputation

| Outcome | 6 weeks post surgery | | |  | 12 weeks post surgery | | |
| --- | --- | --- | --- | --- | --- | --- | --- |
|  | Coefficient | 95% CI | P value |  | Coefficient | 95% CI | P value |
| IKDC | 4.933 | (-1.03, 10.90) | 0.105 |  | 0.465 | (-5.50, 6.43) | 0.879 |
| IKDC (MAX adjusted to 100) | 5.670 | (-1.19, 12.53) | 0.105 |  | 0.535 | (-6.32, 7.39) | 0.879 |
| LEFS | -1.429 | (-6.65, 3.79) | 0.591 |  | -2.776 | (-7.99, 2.44) | 0.297 |
| SF-12 PCS | -2.946 | (-6.56, 0.67) | 0.110 |  | -2.715 | (-6.33, 0.90) | 0.141 |
| SF-12 MCS | 3.814 | (-1.53, 9.16) | 0.162 |  | 1.100 | (-4.25, 6.45) | 0.687 |
| NPRS | 0.214 | (-0.56, 0.98) | 0.586 |  | 0.291 | (-0.48, 1.06) | 0.458 |
| Active Knee Flexion (°) | 5.425 | (-1.02, 11.87) | 0.099 |  | 2.409 | (-4.04, 8.85) | 0.464 |
| Active Knee Extension (°) | -1.238 | (-3.61, 1.14) | 0.307 |  | 0.021 | (-2.35, 2.40) | 0.986 |
| Active ROM (°) | 5.922 | (-2.03, 13.87) | 0.144 |  | 2.831 | (-5.12, 10.78) | 0.485 |
| Passive Knee Flexion (°) | 4.014 | (-3.14, 11.16) | 0.271 |  | 2.793 | (-4.36, 9.94) | 0.444 |
| Passive Knee Extension (°) | -0.961 | (-3.40, 1.48) | 0.440 |  | -0.193 | (-2.64, 2.25) | 0.877 |
| Passive ROM (°) | 4.975 | (-3.28, 13.23) | 0.237 |  | 2.986 | (-5.27, 11.24) | 0.478 |
| IKDC= International Knee Documentation Committee Subjective Knee Form; LEFS: Lower Extremity Functional Scale; SF-12=12-item Short Form Survey; PCS= Physical Component Score; MCS= Mental Component Score; NPRS= Numeric Pain Rating Scale; ROM= Range of Motion | | | | | | | |
